# Conditional ablation of p130Cas/BCAR1 adaptor protein impairs epidermal homeostasis by altering cell adhesion and differentiation

**DOI:** 10.1186/s12964-018-0289-z

**Published:** 2018-11-03

**Authors:** Maria del Pilar Camacho Leal, Andrea Costamagna, Beatrice Tassone, Stefania Saoncella, Matilde Simoni, Dora Natalini, Aurora Dadone, Marianna Sciortino, Emilia Turco, Paola Defilippi, Vincenzo Calautti, Sara Cabodi

**Affiliations:** Department of Biotechnology and Health Science, Molecular Biotechnology Center, Università di Torino, Via Nizza 52, Torino, Italy

**Keywords:** Adaptor proteins, Mouse primary keratinocytes, Cell adhesion, Cell signaling and cell differentiation

## Abstract

**Background:**

p130 Crk-associated substrate (p130CAS; also known as BCAR1) is a scaffold protein that modulates many essential cellular processes such as cell adhesion, proliferation, survival, cell migration, and intracellular signaling. p130Cas has been shown to be highly expressed in a variety of human cancers of epithelial origin. However, few data are available regarding the role of p130Cas during normal epithelial development and homeostasis.

**Methods:**

To this end, we have generated a genetically modified mouse in which p130Cas protein was specifically ablated in the epidermal tissue.

**Results:**

By using this murine model, we show that p130Cas loss results in increased cell proliferation and reduction of cell adhesion to extracellular matrix. In addition, epidermal deletion of p130Cas protein leads to premature expression of “late” epidermal differentiation markers, altered membrane E-cadherin/catenin proteins localization and aberrant tyrosine phosphorylation of E-cadherin/catenin complexes. Interestingly, these alterations in adhesive properties in absence of p130Cas correlate with abnormalities in progenitor cells balance resulting in the amplification of a more committed cell population.

**Conclusion:**

Altogether, these results provide evidence that p130Cas is an important regulator of epidermal cell fate and homeostasis.

**Electronic supplementary material:**

The online version of this article (10.1186/s12964-018-0289-z) contains supplementary material, which is available to authorized users.

## Background

p130Cas is a multifunctional adaptor protein required for embryonic development and is characterized by structural motifs that enable interactions with a variety of signaling molecules and the modulation of pathways controlling cell proliferation, survival, actin cytoskeleton organization and extracellular matrix degradation [1]. p130Cas/BCAR1 expression has been shown to be fundamental for cell transformation and tumor progression in several cancers but also in other diseases [2, 3].

The early embryonic lethality of the germ-line knockout (KO) p130Cas/BCAR1 mouse points out its crucial role during mouse development [4]. To circumvent embryonic lethality in order to investigate the role of p130Cas in mammalian developmental process, we have established a p130Cas tissue-specific knockout mouse line, utilizing Cre transgene under control of the human cytokeratin 14 (K14) gene promoter, which is active in dividing cells of several stratified epithelial tissues, including skin and mammary gland [5].

Epidermal stratification is achieved through two distinct mechanisms. The first one involving the basal cells detachment and transition from the basement membrane to the suprabasal layers. The second mechanism accounts for the asymmetrical cell division of stem cells residing in the basal layer that generate a suprabasal cell that is committed to terminal differentiation but still undergoes a limited number of cell division and a slow cycling cell that remains confined into the basal layers [6–8].

Once detached from the basal membrane, epithelial cells undergo differentiation progressing through a series of stage-specific morphological and biochemical changes leading to the synthesis of the differentiation-specific keratins such as Cytokeratin 1 (K1) and Cytokeratin 10 (K10), as well as involucrin, filaggrin, and loricrin [9, 10]. In in vitro cell culture, elevation of calcium concentrations induces keratinocyte differentiation program characterized by differentiation markers expression, rapid redistribution of cadherins to the membrane and consequent adherens junctions and desmosomes formation, reorganization of the cytoskeleton, polarization and stratification [11].

Analysis of mouse skin tissue in vivo and mouse keratinocyte cultures showed that the absence of p130Cas expression is sufficient to alter epidermal homeostasis. Indeed, epidermal ablation of p130Cas impairs cell-matrix adhesion, increases cell proliferation and expression of terminal differentiation markers both in vivo and in vitro. Interestingly, undifferentiated p130CasKO keratinocyte cultures display features of early commitment to differentiation characterized by E-cadherin cell membrane localization along with an increased tyrosine phosphorylation of E-cadherin and beta-catenin. The alterations in cell proliferation, differentiation and adhesion properties observed in p130Cas null keratinocytes correlate with the expansion of cell population, with reduced clonogenic ability, which are consistent with a differentiation committed phenotype.

## Materials and methods

### Knock-out mice generation

The use of animals was in compliance with the Guide for the Care and Use of Laboratory Animals published by the U.S. National Institutes of Health and approved by the Italian Health Minister (authorization n° 24-2014PR).

C57/BL mice containing loxP sites flanking exon 5 and 6 and the neomycin cassette were generated by first crossing them with flipper (FLPe) and then crossing p130Cas ^f/f^ mice with K14-Cre transgenic animals. Genotypes were determined by PCR screening of tail biopsies by using the following primers: p130CasloxP FW (5’-GATACCTTCTGGGTCTCCTGTACCCC AAGG-3′) and p130CasloxP RW (5’-CCTCTGCTTCCCAAATGCTGGGATCAA AGG-3′) to identify loxP sites flanking p130Cas gene, Cre FW (5’-GGACATGTTCAGGGATCGCCAGGCG-3′) and Cre RW (5’-GCATAACCAGTG AAACAGCATTGCTG-3′) to identify Cre recombinase.

### Cell proliferation, differentiation and ECM-cell adhesion assays

For keratinocyte cell differentiation, 2 mM CaCl_2_ was added to keratinocyte culture medium. For proliferation assays 5 × 10^5^ cells/ml were plated in triplicate on collagen coated 12-well dishes in low calcium medium (minimal essential medium with 4% Chelex treated fetal calf serum (Hyclone), epidermal growth factor (EGF; 10 ng/ml, Collaborative Research, Inc., Cambridge, MA), and 0.05 mM CaC12 (low calcium medium) as described in (Calautti et al.*,*1998). Cells were counted from the first day after plating for three days in triplicate. For cell adhesion experiments, the number of attached cells to the ECM components was evaluated the day after plating.

### Immunoprecipitations

Mouse primary keratinocytes immunoprecipitation experiments were performed as described in (Calautti et al.*,*1998). Briefly, keratinocytes were lysed and quantified. Same extract amounts were incubated ON at 4 °C with antibodies followed by addition of Protein G–agarose beads for 2 h at 4 °C. Immune-complexes were washed four times, eluted in boiling Laemmli sample buffer and separated by SDS-PAGE.

### Western blot analysis

Extracts from different tissues (heart, brain and skin) were prepared from 3-day-old mice. Tissues were lysed with Ultra Turrax in boiling SDS Lysis Buffer (2% SDS, Tris HCl pH 7.5, 0.5 M EDTA), with protease inhibitor cocktail (Roche), and centrifuged at 13000 rpm for 15 min. Primary keratinocytes lysates were prepared by scraping 6-well dishes into 100 μl boiling SDS Lysis Buffer, vortexed and boiled several times. Lysates were subjected to western blotting analysis with the following antibodies: filaggrin, loricrin, keratin 1 and keratin 5 (Covance (Princeton, NJ, USA)); p130Cas, ΔNp63, E-cadherin, beta-catenin and alpha-catenin (BD Transduction Laboratories (San Jose, CA,USA)); tubulin and c-Src (Sigma (St. Louis, MI, USA)); phospho-Erk1/2 MAPKs (Thr202/Tyr204), phospho-Src (Tyr416), phospho-YAP and Yap (Cell Signaling (Danvers, MA, USA)); Erk1/2, CyclinD1, pTyr (Santa Cruz (Palo Alto, CA, USA)). Secondary antibodies were incubated for 1 h at RT, and detection performed with ECL Prime (GE Healthcare, Chicago, IL, USA). Protein band intensities were determined using the Image J software. All comparative images of blots shown are resulting from same exposures of the same membranes.

### Immunohistochemistry and immunofluorescence analysis

For histological analysis, frozen sections from 3-days-old mice dorsal skin were stained with hematoxylin/eosin (H/E). For immunofluorescence staining, sections were fixed in methanol/acetone solution, permeabilized with PBS/0.5% Triton X-100, saturated with blocking buffer for 30′ and incubated with primary antibodies at 4 °C ON followed by 1 h incubation at RT with Alexa Fluor 488 or 568 antibodies (Invitrogen). Images were taken at HCX PL APO CS 40X-63X 1.4 OIL Leica TCS-SP5 II confocal microscope and analysed with LASAF software or at Zeiss Observer Z.1 microscope (× 20/0.50 objective) with the ApoTome module. For PCNA and Ki67 staining (Santa Cruz, (Palo Alto, CA, USA); Novocastra, (Leica, UK)), sections were fixed and stained as above. Staining was detected by peroxidase reaction with DAB. ImageJ software (NIH, Bethesda USA) was used for quantification and/or counting analysis.

### Primary mouse keratinocyte cell culture and treatments

Primary epidermal keratinocytes were isolated from the skin as previously described (Calautti et al., 1998). Briefly, skin was removed from 3-days-old pups; the epidermis was peeled away from the dermis, minced and filtered to release single cells. Keratinocytes (1 × 10^6^ cells/ml) were plated in culture plates coated with collagen and cultured in low calcium medium at 34 °C and 8% CO_2_.

### Colony-forming efficiency (CFE) assays

Colony-forming efficiency (CFE) assays were performed as described in [12], with minor modifications. 10^3^ cells were plated on lethally irradiated feeder layer 3 T3-J2 cells. After 12 days, colonies were fixed, stained with rhodamine-B and scored under a dissecting microscope. Total colonies were calculated as a percentage of total plated cells (colonies number × 100/cell plated number). For second progeny experiments, primary cells isolated from WT and p130CasKO mice were maintained in low calcium condition for 7 days. Then CFE assays were performed with an increased number of cells (5 × 10^4^). Colonies were classified based on morphological parameters [12, 13].

### Dispase treatment of mouse primary keratinocytes

Dispase treatment was performed as described in [14]. Briefly, duplicate 60-mm dishes of confluent keratinocyte cultures in low or high calcium medium were washed twice in PBS and incubated in 2 ml dispase solution in PBS (2.4 units/ml; Roche) at 34 °C. Cells were analyzed under the microscope at 5-min intervals, for 35 min. After 35 min, cells were scraped, then washed twice in PBS and centrifuged. Cell pellets were resuspended and counted as single cells with an hemocytometer. Results are expressed as percentage of dispase released cells over the total number of cells.

### Statistical analysis

Statistical analyses were performed with Student t test or two-way analysis of variance (ANOVA, Bonferroni posttest analysis) for comparison of two or multiple groups, respectively, using Prism 5.0 (GraphPad, San Diego, CA). Mann-Whitney nonparametric test was applied when quantified results do not follow normal distribution. Differences with *P*-values less than 0.05 were considered statistically significant.

## Results

### Generation of mice with epidermis-restricted deletion of the p130Cas/BCAR1 gene

**Fig. 1:**
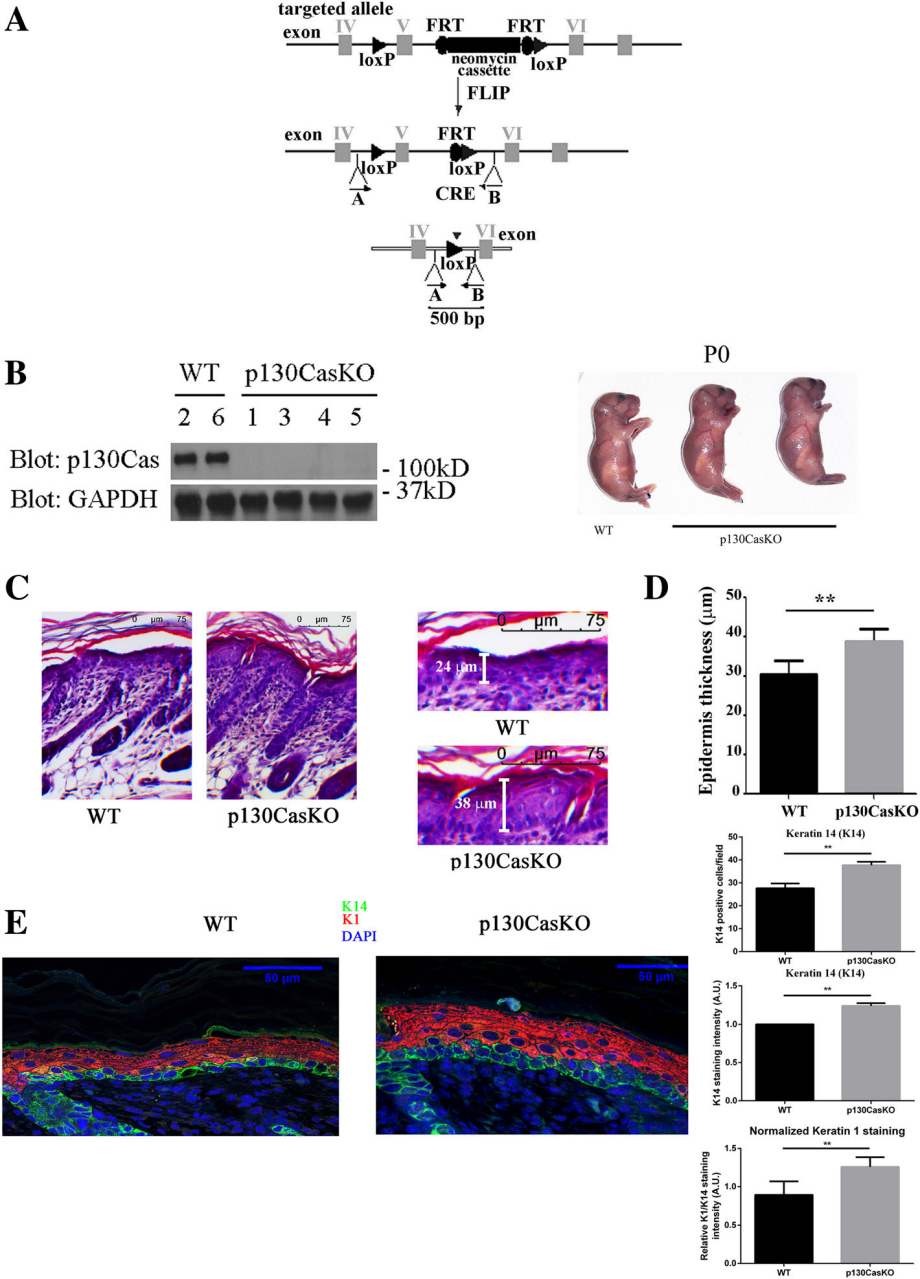
Conditional deletion of p130Cas in the mouse epidermis. (**a**) Schematic representation of p130Cas/BCAR1 gene targeting strategy. (**b**) Epidermis isolated from WT (mice 2, 6) and p130CasKO (mice 1, 3, 4 and 5) 3 days old pups were lysed and cell extracts were subjected to western blotting protein analysis. Representative images of WT and p130CasKO mice at birth (PO) (right panels). (**c**) Representative haematoxylin/eosin stained sections from WT and p130CasKO skins of 3 days old pups (10X) (left panels). Higher magnification insets of representative haematoxylin/eosin stained section from WT and p130CasKO skins (20X) (right panels). (**d**) Quantification of epidermis thickness in WT and p130CasKO. The data represented the mean ± S.D. from 10 WT and 10 p130CasKO mice (**p < 0.01). (**e**) Representative images of K14 and K1 fluorescence staining of WT and p130CasKO 3-day-old pups skin (40X) (left panel). Quantification of immunofluorescent stainings is shown in the right panels. K1 staining intensity was normalized for K14 staining intensity. The data represented the mean ± S.D. from 6 WT and 6 p130CasKO mice (**p < 0.01) (field equal to 200 μm)

To conditionally ablate p130Cas expression in the epidermis, we employed the Cre-LoxP system. p130Cas fl/fl mutant mice harbors a construct in which exons 5 and 6 of the p130Cas gene were flanked with Cre-lox recombination (loxP) sites (Fig. 1a). Mice were first mated with flipper (FLPe) mice to remove the neo-cassette and one FLPe recognition target (FRT) site, leaving behind the two loxP sites. p130Cas^f/f^ mice were bred with transgenic mice expressing Cre recombinase under the control of the human K14 promoter. This promoter is active in dividing cells of several stratified epithelia, such as skin, mammary gland and thymus (Jonkers et al., 2001). To evaluate the absence of p130Cas in the Knock-Out (KO) mice, western blot analyses were performed on extracts of skin isolated from 3-day-old p130CasKO mice (Fig. 1b) and of different tissues, such as brain, heart and thymus (Additional file 1: Figure S1). This analysis confirmed that Cre recombination occurred in the epidermis and in thymus as reported, but not in other tissues. p130CasKO pups were born at the expected Mendelian ratio, were viable and fertile, and no significant differences were observed at birth in growth or macroscopic defects of the skin relative to control littermates lacking Cre recombinase (Fig. 1b).

### p130Cas deficiency alters the balance between epidermal cell proliferation and differentiation

Histological examination of the epidermis of 3-day-old p130CasKO and Wild-Type (WT) pups revealed a significant epidermal thickening in mutant mice, indicating altered skin morphogenesis in the absence of p130Cas (Fig. 1c and d). We also analyzed the expression of K1, an early differentiation marker typical of the spinous suprabasal epidermal layer as well as of K14, whose expression is confined in basal undifferentiated epidermal layer under homeostatic physiological conditions [15]. K1 staining was significantly enhanced in the suprabasal layers of p130CasKO skin as compared to WT counterparts. Moreover, we also observed a higher number of K14 positive cells in the suprabasal layers of p130CasKO mice, suggestive of abnormalities in the balance between keratinocyte proliferation and differentiation (Fig. 1e and Additional file 1: Figure S2).

**Fig. 2:**
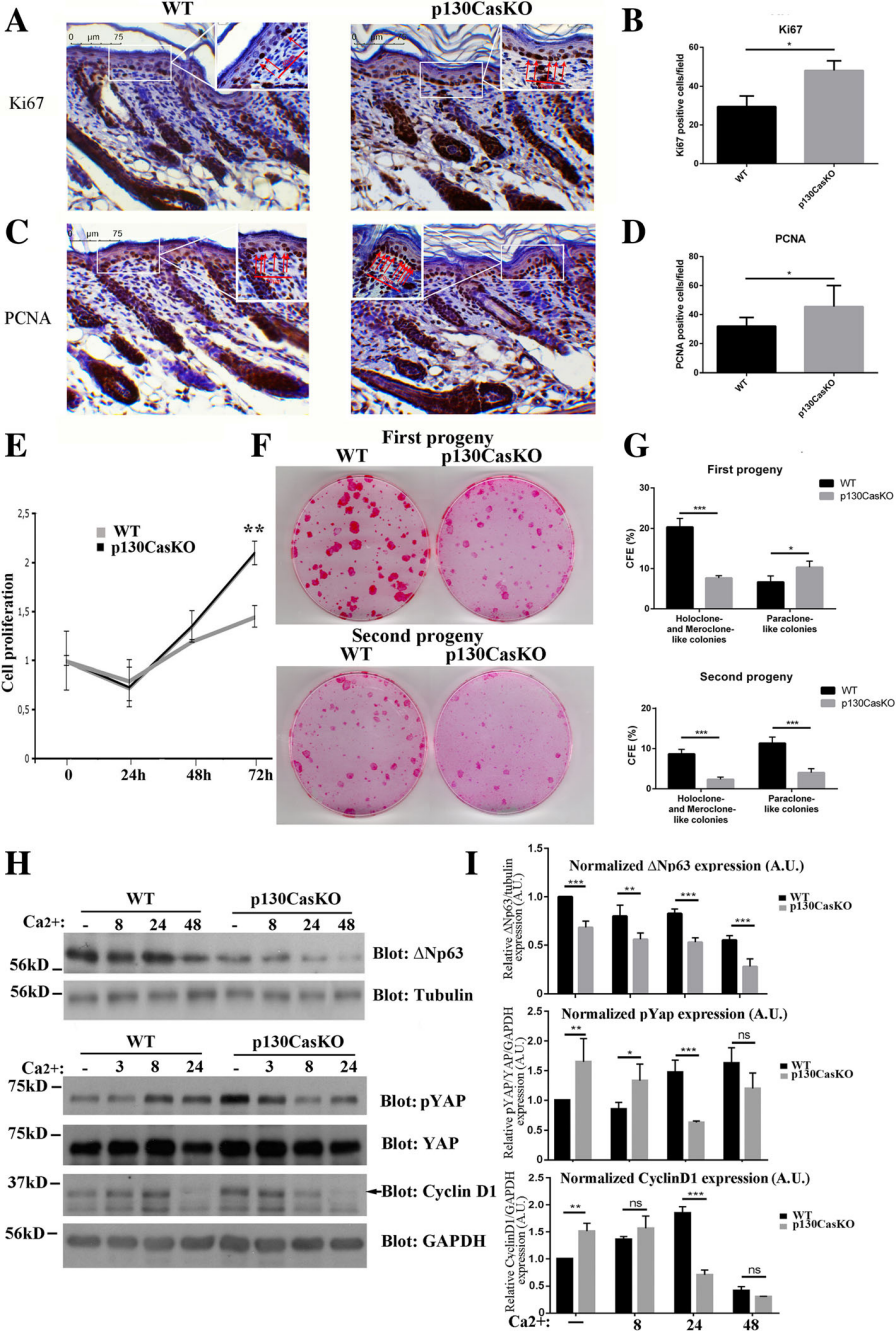
p130Cas deletion alters the balance between epidermal proliferation and differentiation. (**a**, **b**) Representative images of Ki67 staining from WT and p130CasKO of 3-day-old pups skin (20X). Quantification was performed by counting Ki67 basal positive cells on the entire images (field equal to 350 μm). The data represented the mean ± S.D. from 10 WT and 10 p130CasKO mice (*p < 0.05). Inset were taken at 100X magnification. (**c**, **d**) Representative images of PCNA staining from WT and p130CasKO of 3-day-old pups skin (20X). Quantification was performed by counting PCNA basal positive cells on the entire images (field equal to 350 μm). The data represented the mean ± S.D. from 10 WT and 10 p130CasKO mice (*p < 0.05). Inset were taken at 100X magnification. (**e**) WT and p130CasKO keratinocytes were plated in triplicate on collagen coated dishes and counted every day for 72 h. Data are expressed as mean ± S.D. of results from three independent experiments (**p < 0.01). (**f**) Representative images of first and second progeny of WT and p130CasKO keratinocytes cultured under clonogenic conditions for 12 days, followed by rhodamine-B staining. (**g**) SEM of colonies of the indicated sizes. 100 colonies were counted from duplicate plates and classified based on morphological parameters. Data represent two independent experiments. (**h**) Untreated and calcium-treated WT and p130CasKO keratinocytes extracts blotted for ΔNp63 (Tubulin as loading control), phospho-YAP, YAP, Cyclin D1 (GAPDH as the loading control). (**i**) Densitometric analysis of protein levels of at least three independent experiments is shown (*p < 0.05, p** < 0.01, ***p < 0.001)

To determine whether the epidermal thickening and suprabasal K14^+^ cells observed in p130Cas-deficient mice correlate with increased keratinocyte proliferation, we assessed the expression of Ki67 and PCNA proliferative markers. As shown in Fig. 2a-d and in Additional file 1: Figure S3, Ki67 and PCNA were significantly increased in the basal layer of p130CasKO mice compared to WT, indicating an increased tendency of mutant keratinocytes to proliferate. Interestingly, PCNA staining was detected in a significant amount of suprabasal cells at both embryonic day 18.5 and P3 in p130CasKO epidermis (Additional file 1: Figure S5).

To provide insights into the control of p130Cas-dependent skin homeostatic processes, in vitro culture of primary keratinocytes isolated from 3 days-old pups were established [16]. In this in vitro model, proliferation assays were performed by counting every day for 72 h WT and p130CasKO keratinocytes plated on collagen coated dishes. The proliferative curve shown in Fig. 2e indicates that p130CasKO keratinocytes possess higher proliferation rates than WT keratinocytes.

Since epidermal stratification involves delamination and asymmetrical cell division of basal cells [7, 8], and we observed a higher percentage of K14 positive cells in the suprabasal layers of the p130CasKO, we hypothesized that p130Cas deletion might impact progenitor cells commitment toward differentiation. Consistently, we found that freshly isolated p130CasKO primary keratinocytes have an overall reduced clonogenic capacity with a strong reduction of colonies displaying holoclone- and meroclone-like morphology, which was also maintained in second-generation clonogenic assays (Fig. 2f and g). Overall, these data are consistent with an anticipated commitment of p130CasKO progenitor cells to differentiation. Accordingly, the levels of ΔNp63α, a transcription factor governing keratinocyte stem cell fate [17], were found reduced in p130CasKO in undifferentiated conditions compared to WT cells, and further diminished upon differentiation induction triggered by elevation of the extracellular calcium concentration (high calcium medium) (Fig. 2h, i and Additional file 1: Figure S4A). Consistently, p63 expression was found decreased in p130CasKO epidermis at both E18.5 (mouse embryonic day 18.5) and P3 (Additional file 1: Figure S5). Moreover, inactivating phosphorylation of YAP, a transcriptional activator implicated in skin homeostasis control [18, 19], was found up-regulated in p130CasKO keratinocytes compared to WT cell under basal conditions. Moreover, although the YAP protein levels did not differ significantly between genotypes in response to calcium (Additional file 1: Figure S4B), the kinetics of YAP phosphorylation indicated an opposite trend between WT and p130CasKO keratinocytes, with an increase in the former and a decrease in the latter cells (Fig. 2h and i). In newborn skin, YAP expression was detected in few basal cells in both WT and mutant mice as previously reported [19, 20]. Notably, p130CasKO epidermis contained a significant amount of nuclear YAP in isolated suprabasal cells compared to WT controls (Additional file 1: Figure S5), and a similar trend was also observed in mutant mice at E18.5 (data not shown). Cyclin D1 expression in vitro was more elevated in proliferating p130CasKO as compared to WT cells (Fig. 2h and i). Overall, these data suggest a role for p130Cas in the maintenance of epidermal homeostasis by promoting keratinocyte quiescence and preventing commitment toward differentiation.

**Fig. 3:**
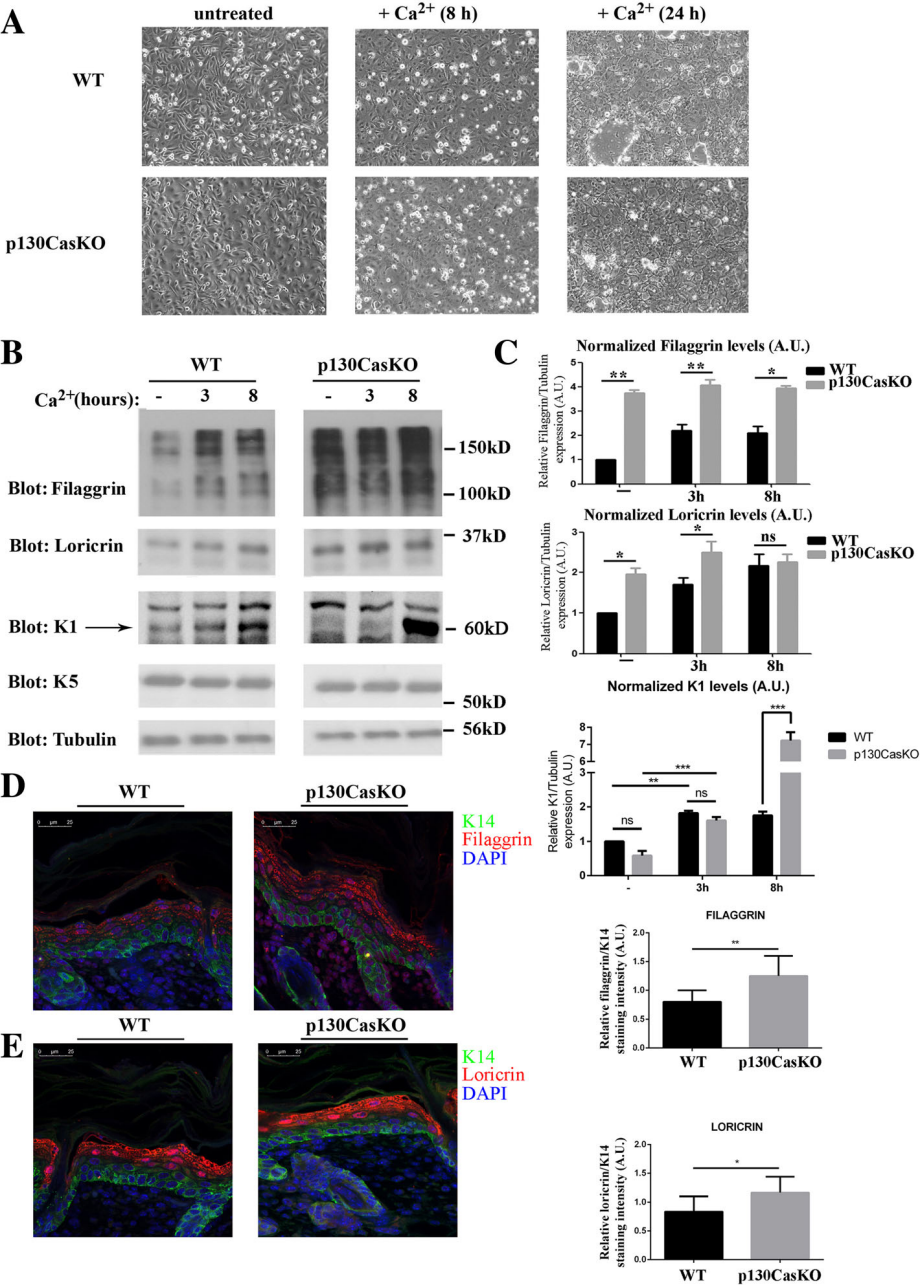
p130Cas loss affects keratinocytes differention. **(a)** Representative images of confluent WT and p130CasKO keratinocytes in low calcium condition and after 8 and 24 h of calcium treatment (20X magnification). (**b**) Western blot analysis of early and late differentiation markers of WT and p130CasKO keratinocytes either untreated or calcium treated. (**c**) Densitometric analysis of protein levels of at least three independent experiments is shown (p* < 0.05, **p < 0.01). (**d**) Representative images and quantification of filaggrin fluorescence staining of WT and p130CasKO 3-day-old pups skin (left and right panel, respectively) (40X). (**e**) Representative images and quantification of loricrin fluorescence staining of WT and p130CasKO 3-day-old pups skin (left and right panel, respectively) (40X). Data are expressed as mean ± S.D. of three independent experiments

The increased proliferation rates observed in p130CasKO cells coupled with their reduced clonogenic capacity suggest that these cells may be more prone to engage the terminal differentiation program. To verify this hypothesis, we compared keratinocytes isolated from WT and mutant mice under undifferentiated conditions or during calcium-induced differentiation. Upon calcium switch, keratinocytes cultures acquire a squamous morphology, become stratified, form desmosomes and adherens junctions, undergo growth arrest and express markers typical of suprabasal epidermal layers. In undifferentiated conditions p130CasKO keratinocytes display a flat and round-shaped morphology compared to wild-type cells. Upon differentiating conditions, p130CasKO cells appear bigger with less evident cell-cell borders (Fig. 3a). Notably, under low calcium culture conditions, p130CasKO cells display a higher expression of granular layer differentiation markers (filaggrin and loricrin) but not spinous layer marker (K1) (Fig. 3b and c). Upon differentiating stimulus, an expected induction of both early and late differentiation marker expression in WT keratinocytes was observed, whereas p130CasKO cells displayed a robust K1 induction after calcium stimulation which was consistent with the increased K1 expression observed in vivo (Fig. 1e). Moreover, although the absolute level of filaggrin and loricrin were overall increased in mutant cells, the levels of these proteins did not further increase upon calcium treatment (Fig. 3b and c). Consistently, filaggrin and loricrin expression was also increased in the intact epidermis of p130CasKO mice (Fig. 3d and e and Additional file 1: Figure S6).

Overall, these data indicate that p130Cas deficiency affects keratinocytes proliferation and differentiation both in vitro and in vivo and renders the cells more prone to engage differentiation.

### p130Cas is required for proper ECM and cell-cell adhesion

**Fig. 4:**
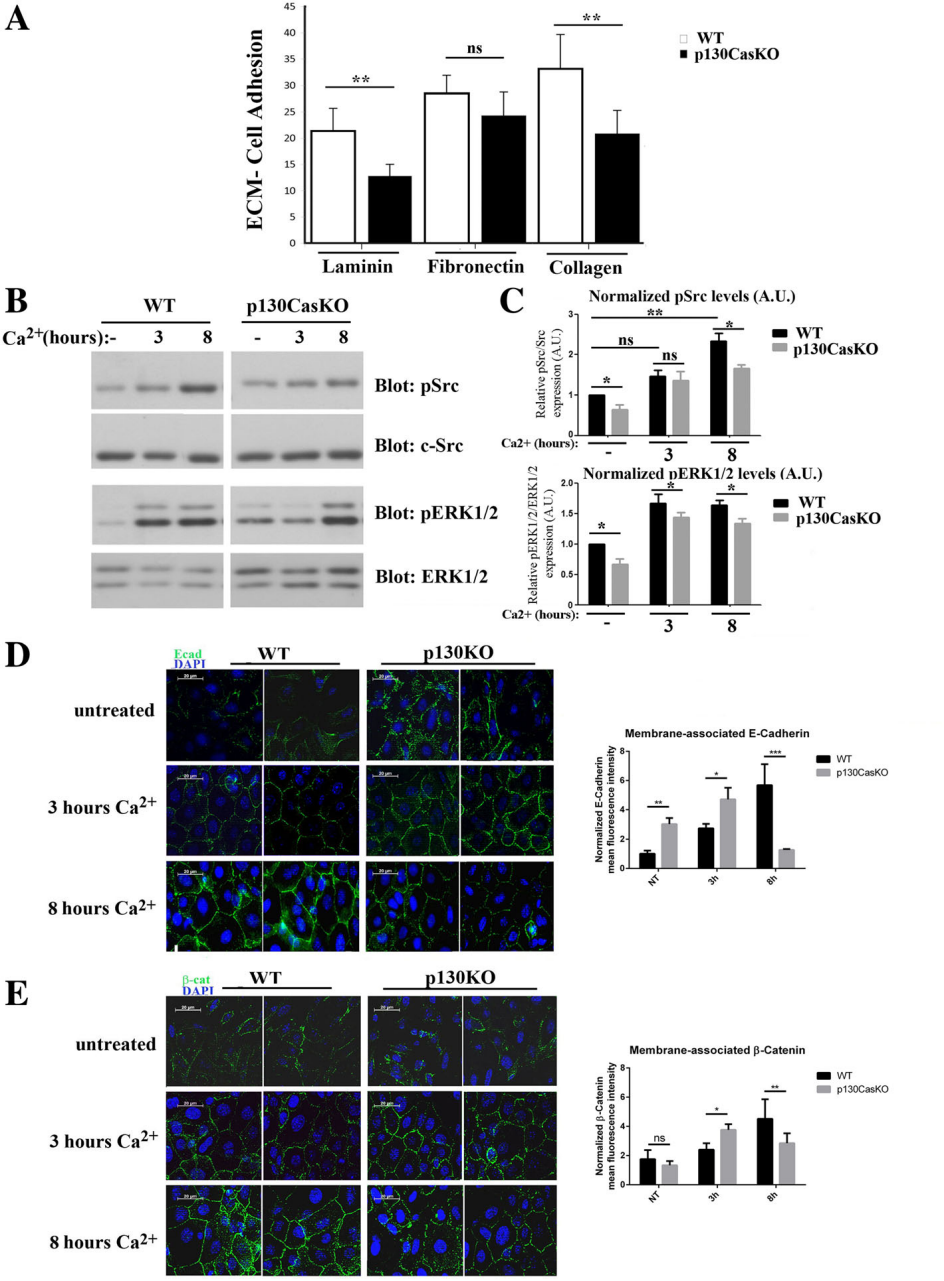
p130Cas is required for proper ECM and cell-cell adhesion. (**a**) Adhesion quantification of freshly isolated WT and p130CasKO primary keratinocytes on laminin, fibronectin and collagen after 12 h of cell adhesion. Data are expressed as mean ± S.D. of five independent experiments (**p < 0.01). (**b**) Western blotting analysis for phosho-Src (pSrc), c-Src, phospho-ERK1/2 MAPKs (pERK1/2) and ERK1/2 MAPKs from confluent untreated and calcium-treated WT and p130CasKO keratinocytes. (**c**) Densitometric analysis of protein levels of at least three independent experiments is shown (p* < 0.05, **p < 0.01). Activation of Src and ERK1/2 MAPKs was normalized on c-Src and ERK1/2 levels, respectively, as loading control. (**d**, **e**) Representative images of E-cadherin (**d**) and beta-catenin (**e**) stainings in untreated and calcium-treated WT and p130CasKO keratinocytes. DAPI stained nuclei (63X magnification) (left panels). Quantification of immunofluorescent staining of membrane-associated E-cadherin and beta-catenin is shown in the right panels. The data represented the mean ± S.D. of 4 independent experiments (*p < 0.05, **p < 0.01, ***p < 0.001) (field equal to 50 μm)

A critical step in the differentiation process of all epithelial cells is cell detachment from the basement membrane, mostly dependent on modification in integrin-matrix interactions. Indeed, activation of beta1 integrins in normal keratinocytes abrogates differentiation while inhibition of integrin downstream signaling promotes keratinocytes differentiation [21–23]. Several lines of evidence have placed p130Cas as an important modulator of signals emanating from integrins and ECM [2]. Therefore, to determine whether the altered differentiation process observed in p130CasKO is due to aberrant ECM-keratinocyte cell adhesion, we performed keratinocyte cell adhesion assays to fibronectin, laminin and collagen ECM components. Specifically, p130CasKO keratinocytes display reduced adhesion to both collagen and laminin as compared to WT cells (Fig. 4a), indicating that p130Cas deficiency in keratinocytes leads to alterations in cell adhesion to basement membrane components. The ECM-cell adhesion reduction observed in p130KO keratinocytes may reflect an aberrant expression of integrin receptors in the basal layer of the mouse epidermis. To test this possibility, we evaluated integrin receptor beta1 and beta4 protein expression levels in keratinocytes derived from WT and KO mice. However, neither beta1 nor beta4 integrin protein levels were affected by p130Cas ablation (Additional file 1: Figure S7). To evaluate whether the observed cell adhesion reduction upon p130Cas deletion may result from defective integrin signaling, we tested whether phosphorylation of Src was altered in p130CasKO keratinocytes compared to WT cells. Src kinase activation is one of the early event associated with integrin engagement to the ECM and is required for p130Cas phosphorylation [24]. As shown in Fig. 4b and c, phosphorylation of Src was significantly reduced in p130CasKO keratinocytes even in low calcium medium, indicating that integrin downstream signaling is impaired. Moreover, ERK1/2 MAPKs activation upon integrin clustering has been correlated to cell decision to undergo differentiation. Indeed, in absence of differentiative stimuli, the levels of ERK1/2 MAPKs activity reflect the capacity of keratinocytes to undergo differentiation [21, 25–27]. Consistently, the deletion of p130Cas in the basal layer impairs integrin signaling resulting in lower activation of MAPKs both in undifferentiated and differentiated condition (Fig. 4b and c).

These results suggest that the absence of p130Cas in basal keratinocytes leads to an alteration in integrin signaling, reflected by an aberrant basal membrane adhesion and a commitment to cell differentiation.

In addition to integrin-cell adhesion, E-cadherin cell-cell dependent adhesion has a crucial role during keratinocyte differentiation. To determine whether alterations in E-cadherin localization and/or activation are implicated in the abnormal cell differentiation of p130Cas-null keratinocytes, we performed E-cadherin and beta-catenin immunofluorescence analysis of insoluble-detergent fractions in confluent WT and mutant primary keratinocytes cultures either kept undifferentiated in low calcium medium or at different times following 2 mM calcium switch. Surprisingly, E-cadherin appeared recruited at the cell membrane in p130CasKO keratinocytes already in low calcium medium while in WT cells, it localized at the cell membrane only after the calcium switch, as expected. (Fig. 4d and e). Interestingly, this increased recruitment of E-cadherin and beta-catenin at the cell membrane, was even more pronounced at early times after calcium treatment in p130CasKO cells. In fact, after 3 h of calcium treatment, p130Cas KO keratinocytes show an almost complete sealing of adjacent cell membranes of juxtaposed cells while WT keratinocytes as reported previously [14, 28, 29] were found in the “zipper-like” stage. However, at later times (8 h), E-cadherin and beta-catenin were found decreased at the cell borders of mutant cultures. These data suggest abnormalities in the ordered assembly of E-cadherin-dependent adhesive structures in the absence of p130Cas.

## p130Cas is required for cell-cell adhesion dynamics

**Fig. 5:**
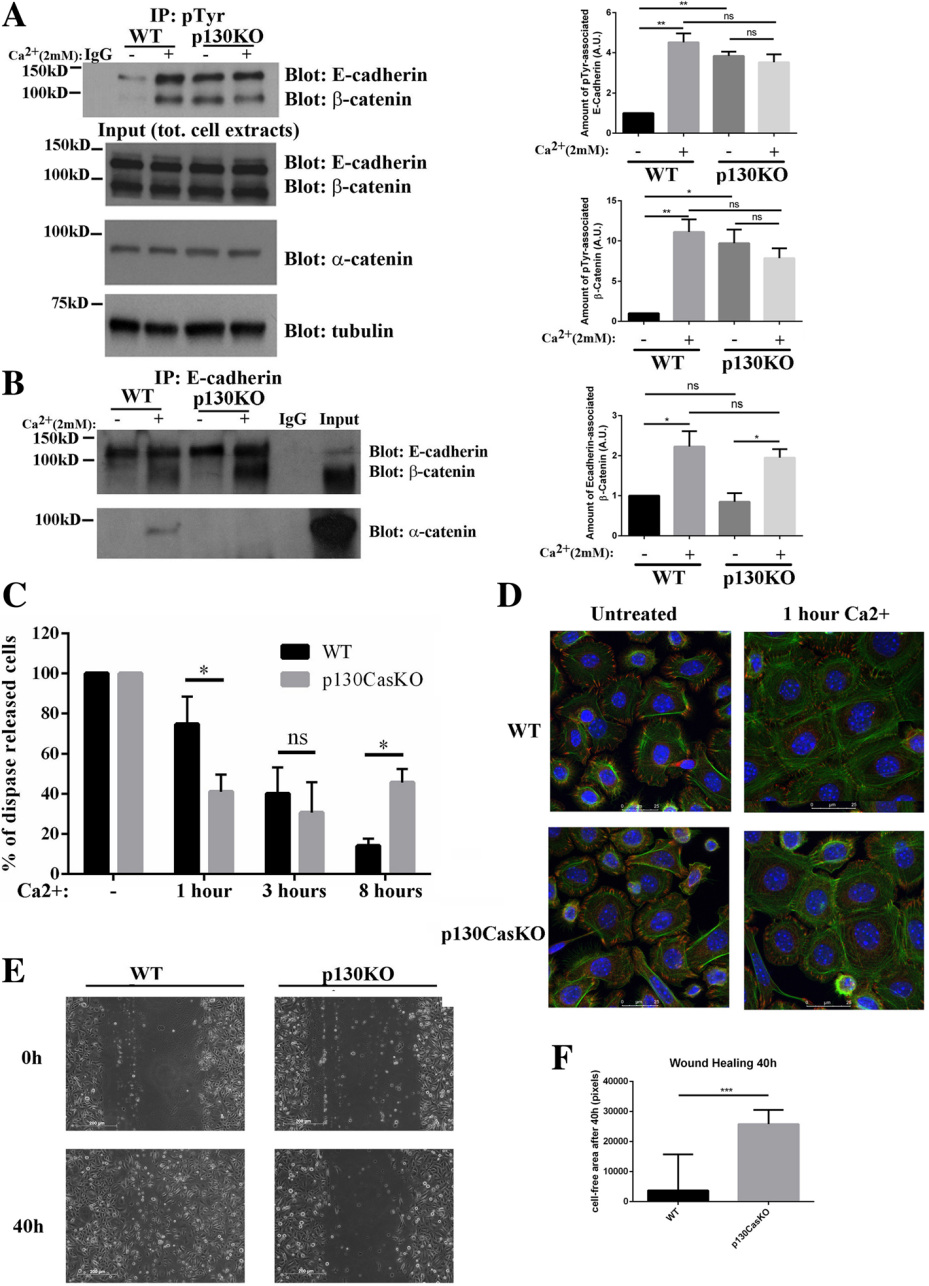
p130Cas impairs cell-cell adhesion dynamics. (**a**) Phospho-tyrosine immunoprecipitates from confluent untreated and calcium-treated WT and p130CasKO keratinocytes blotted with E-cadherin and beta-catenin antibodies. IgG indicates the immunoprecipitation control. Cell extracts are shown at the bottom (left panels). Densitometric analysis of protein levels of at least three independent experiments is shown in the right panels (p* < 0.05, **p < 0.01). (**b**) E-cadherin immunoprecipitates from the same extracts as in (**a**) blotted with alpha-catenin antibodies (left panels). Densitometric analysis of protein levels of at least three independent experiments is shown on the right panel (p* < 0.05). (**c**) Triplicate samples of primary keratinocytes from WT of p130Cas KO mice were kept under low calcium condition or treated with calcium for 1, 3 and 8 h. Data are expressed as percentage of single cells released by mechanical disruption after dispase treatment versus total number of cells recovered after subsequent treatment of the same sample with trypsin. (**d**) Representative images of actin (green) and vinculin (red) staining of WT and p130CasKO keratinocytes in LCM or 1 h after calcium treatment (63X). Experiments in **c** and **d** were performed three times. (**e**) Representative images of wound healing experiments at time 0 and after 48 h (10X) (left panel), and relative quantification (right panel) by using nonparametric Mann-Whitney test (***p < 0.001). Data are expressed as median ± range of three independent experiments

Tyrosine phosphorylation of adherens junction components is a hallmark of keratinocytes differentiation [14, 28]. Therefore, we investigated whether p130Cas ablation in the epidermis also affects tyrosine phosphorylation dynamics of E-cadherin/beta-catenin complexes. For this reason, we determined the amount of E-cadherin/beta-catenin complexes that could be recovered after immunoprecipitation with anti-phosphotyrosine antibodies (p-Tyr) both in low and high calcium condition (8 h). In undifferentiated conditions, increased E-cadherin and beta-catenin tyrosine phosphorylation was observed in p130CasKO immunoprecipitates compared to WT. While in WT keratinocytes calcium addition increased the amounts of E-cadherin and beta-catenin precipitated by p-Tyr antibodies, as expected, in p130CasKO cells the levels of E-cadherin/beta-catenin in complex with p-Tyr were similar in both low- and high calcium conditions (Fig. 5a). Interestingly, in p130CasKO cells alpha-catenin failed to associate to E-cadherin/beta-catenin complexes in high calcium conditions, while no differences in E-cadherin/beta-catenin association were observed in the absence of p130Cas (Fig. 5b).

To evaluate the presence of functional defects in cell-cell junctions caused by p130Cas deletion, we performed a dispase treatment as described in [14]. Indeed, a lack of cohesive strength may be revealed under these conditions, when keratinocytes lose attachment to their support and are connected to each other only through direct intercellular contacts. As shown in Fig. 5c, after 1 h of calcium treatment, p130CasKO keratinocytes display a significantly enhanced strength of cell-cell junctions after dispase treatment compared to WT cells. Interestingly, the anticipation of cell-cell adhesion observed in p130CasKO keratinocytes were reverted at later time points where cells display reduced strength of cell-cell adhesion after 8 h of calcium treatment. However, at even later time points (24 h) no significant changes in dispase sensitivity were observed between WT and p130CasKO keratinocytes, indicating a transient alteration of the adherence junction dynamics in vitro. Interestingly, a similar transient defect of E-cadherin localization at the cell borders, was observe at E18.5 in mutant embryos and this coincided with partial loss of epidermal barrier function (Additional file 1: Figure S8). However, in mutant newborn mice, these defects were fully recovered as they did not display either defects of E-cadherin localization or alterations of barrier function (data not shown).

Moreover, to verify whether the alterations in cell-cell adhesion associate with alterations in actin cytoskeleton, WT and p130CasKO primary keratinocytes in low calcium and after 1 h of calcium treatment, were stained with phalloidin and vinculin. The results shown in Fig. 5d, indicate that in low calcium condition actin fibers are more densely packed in p130CasKO compared to WT cells. After 1 h of calcium exposure, p130Cas KO keratinocytes show increased cortical actin at cell-cell borders while in WT keratinocytes periferal actin is mainly found “zipper-like” structures.

Under low calcium conditions, p130CasKO keratinocytes show a diffused, not-polarized vinculin staining that differs from WT cells in which vinculin is concentrated in focal points. To verify whether this changes correlate with a different cell migratory behavior, we performed in vitro wound healing experiments and found a significant reduction of p130CasKO keratinocytes in wound closure (Fig. 5e and f).

**Fig. 6:**
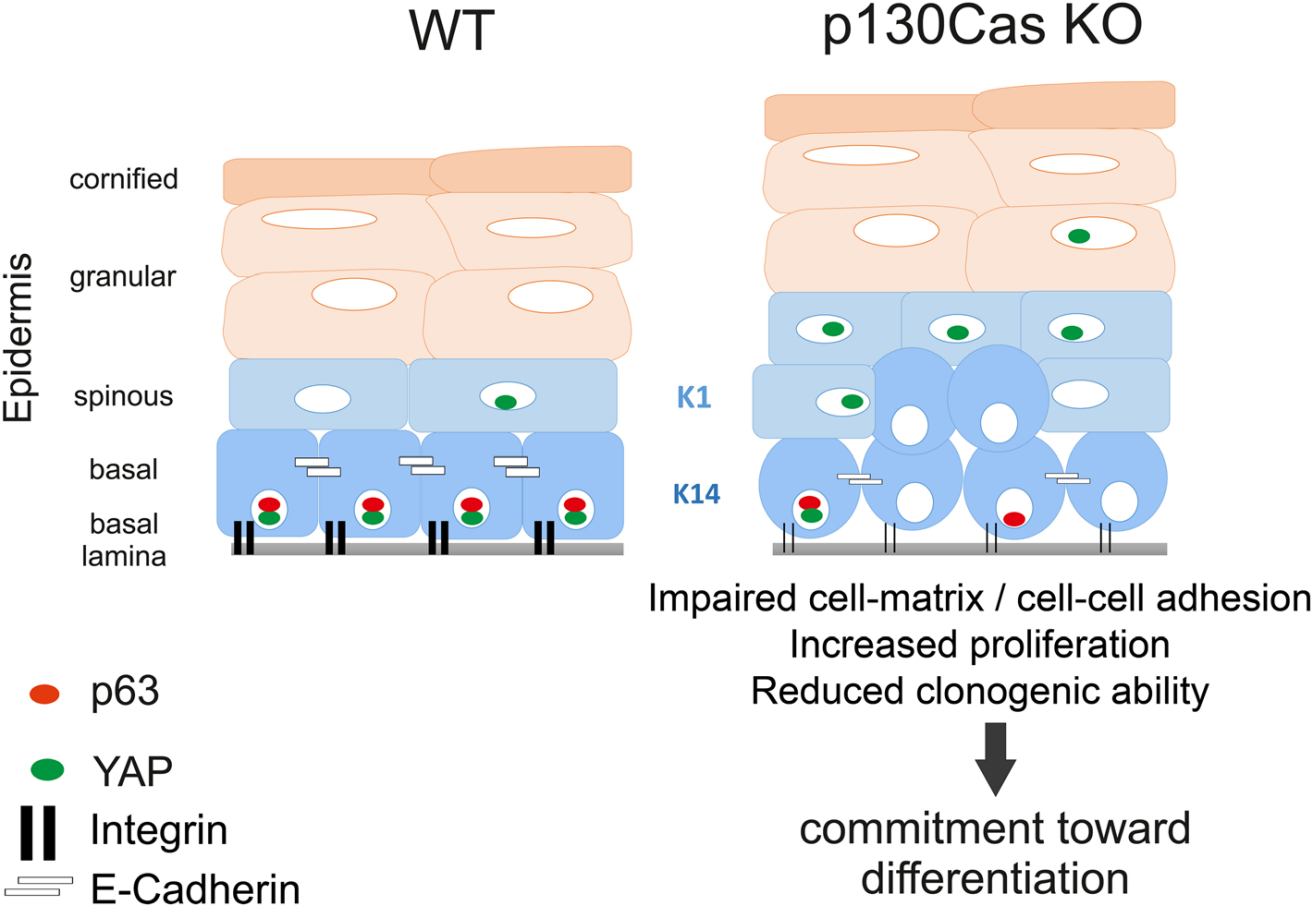
Schematic model of p130Cas-dependend alterations in skin homeostasis. p130Cas loss in basal keratinocytes leads to an alteration in integrin and cadherin adhesive platforms, reflected by reduced ECM attachment and aberrant formation of cell-cell junctions. These signaling hubs are important regulators of p63 and YAP, key determinants of keratinocyte commitment to differentiation. Consistently, epidermal p130Cas loss induces an amplification of premature differentiation-committed keratinocytes characterized by increased proliferation, reduced basal p63 expression and ectopic suprabasal YAP nuclear localization. The altered balance between keratinocyte proliferation and differentiation promotes epidermal thickening in mutant mice

In conclusion, these data indicate that absence of p130Cas alters the correct execution of the keratinocyte differentiation program, by anticipating events connected to adhesion and/or cytoskeleton dynamics that are typical of early stages of differentiation (Fig. 6).

## Discussion

In this study we show that the conditional deletion of p130Cas/BCAR1 gene in the epidermis impacts on keratinocyte biology. Newborn mice lacking p130Cas have epidermal tissue characterized by increased thickness coupled with an increased proliferation in the basal layer compartment and increased expression of K1, filaggrin and loricrin differentiation markers in the suprabasal epidermal layers. Primary keratinocytes derived from these mice also show enhanced proliferation and increased expression of filaggrin and loricrin when kept under standard undifferentiated conditions. p130Cas-null primary keratinocytes also display defects in ECM and cell-cell adhesion. The coexistence of enhanced proliferation coupled with premature differentiation is consistent with an amplification of differentiation-committed progenitor cell populations.

In the epidermis, the stem cell compartment undergoes asymmetrical cell division generating committed, differentiating progenitor cells that are further subjected to few rounds of cell division after which they undergo terminal differentiation by moving upward [8, 30]. The prerequisite to generate these progenitor cells is loss of integrin-based adhesion. Several reports have indeed demonstrated that reduced integrin expression or signaling in adherent cells triggers terminal differentiation and that increased activation of beta 1 integrin prevents terminal differentiation [22, 25, 31]. A key downstream signaling through which integrin-mediated adhesion can mediate keratinocytes differentiation is the ERK1/2 MAPK pathway. Specifically, it has been demonstrated that beta 1-induced activation of ERK1/2 MAPK pathway suppresses terminal differentiation [21, 25–27]. Interestingly, we demonstrate that the absence of p130Cas in keratinocytes impairs Src and ERK1/2 MAPK signaling that is known to be a crucial downstream effector of integrin-based cell-matrix adhesion. Thus, our data are consistent with a loser attachment of cells to the ECM and subsequent impairment in integrin-downstream signaling being important determinants of p130CasKO premature differentiation.

Our data also indicate that p130Cas KO keratinocytes have overall reduced clonogenic abilities, consistent with the possibility that p130Cas plays a role in maintaining stem/progenitor cells in the undifferentiated compartment while its absence promotes the exit of cells toward differentiation.

This possibility is further supported by the observation that ΔNp63 expression, whose downregulation is an important determinant of commitment of keratinocytes toward differentiation [32], is reduced both in p130CasKO cultured cells and epidermis in vivo. Additionally, several reports indicate also that YAP1 is an essential regulator of epidermal maintenance and skin homeostasis [20, 33]. It has been shown that dephosphorylation of YAP and its consequent nuclear translocation regulate the balance between stem cell proliferation and differentiation [34]. Notably, our data indicate that YAP phosphorylation is enhanced in p130CasKO keratinocytes under culture conditions known to maintain the undifferentiated state, with a parallel anticipation of differentiation marker expression. Interestingly, p130CasKO epidermis contained significant amounts of nuclear YAP in isolated suprabasal cells, condition reminiscent of suprabasal YAP expression of alpha-catenin KO epidermis, which also displays increased basal and suprabasal cell proliferation [33].

Moreover, both ΔNp63 and YAP functions in skin homeostasis are linked to both cell-matrix and cell-cell based adhesion [20, 32–36]. Consistently, our data indicate also that loss of p130Cas has unexpected effects on keratinocyte cell-cell adhesion during differentiation.

Adherence junctions are constantly remodeled during epidermal cell differentiation and stratification [9, 37]. The epithelial AJ transmembrane core consists of E-cadherin, whose extracellular domain binds calcium ions to mediate homophilic interactions between neighboring cells [29]. The E-cadherin intracellular domain binds directly to beta-, gamma- catenins, which in turn bind to alpha-catenin, tethering adherence junctions with the underlying actin cytoskeleton, thus providing adhesive strength. Our results indicate that in p130CasKO keratinocytes, E-cadherin is prematurely recruited at cell membrane even in the absence of calcium-induced cadherin engagement. Moreover, pTyr-immunoprecipated E-cadherin/beta-catenin complexes, which are normally found in WT cells in differentiating conditions [14, 16], are robustly detected in p130CasKO cells already under proliferating/undifferentiated conditions, further indicating an anticipation of differentiation-related events occurring in mutant cells. However, our data suggest also that the anticipated engagement of cell-cell adhesion in p130CasKO keratinocytes is not sustained at later times of differentiation, as indicated by dispase-based cell-cell adhesion assay. This may depend, at least in part, to the impairment of alpha-catenin association with E-cadherin/beta-catenin complexes in p130CasKO cells, with a subsequent weakening of the link between adherens junction components and the actin cytoskeleton. It has been proposed that actin cytoskeleton assembly both in response to integrin-mediated and cell-cell adhesion regulates terminal differentiation [38, 39]. Of note, a relevant, albeit transient, impairment in epidermal barrier function was detected in embryonic mutant epidermis that was paralleled with a decrease of E-cadherin at cell borders and enhancement of cell proliferation, a phenotype reminiscent of the one caused by E-cadherin ablation in the epidermis [40].

## Conclusions

We propose that p130Cas by regulating both integrin-dependent and cell-cell adhesion at the cell differentiation switch, finely tunes the balance between epidermal cell proliferation and differentiation by integrating multiple signaling pathways that are essential for epidermal morphogenesis and homeostasis (see Fig. 6), reinforcing the emerging view that in the epidermis cell-cell and cell-ECM adhesion are tightly linked in their regulatory mechanisms.

## Additional file

Additional file 1: **Figure S1.** Skin specific deletion of p130Cas/BCAR1 gene. **Figure S2.** Keratin1 staining. **Figure S3.** Dot plot quantification of Ki67 and PCNA. Fig. S4 ΔNp63 and YAP expression. **Figure S5.** Epidermal PCNA, YAP and ΔNp63 expression in WT and p130CasKO mice. **Figure S6.** Dot plot quantification of loricrin and filaggrin staining. **Figure S7.** Deletion of p130Cas does not alter expression of β1 and β4 integrins. **Figure S8.** Epidermal p130Cas loss alters E-cadherin expression and barrier formation. (DOCX 6794 kb)

## Abbreviations

E18.5: mouse embryonic day 18.5
K1: Cytokeratin 1
K10: Cytokeratin 10
K14: Cytokeratin 14
K5: Cytokeratin 5
KO: Knock-Out
pTyr: phosphoTyrosine
WT: Wild-ytype
