# Correction to: Conditional ablation of p130Cas/BCAR1 adaptor protein impairs epidermal homeostasis by altering cell adhesion and differentiation

**DOI:** 10.1186/s12964-018-0296-0

**Published:** 2018-11-26

**Authors:** Maria del Pilar Camacho Leal, Andrea Costamagna, Beatrice Tassone, Stefania Saoncella, Matilde Simoni, Dora Natalini, Aurora Dadone, Marianna Sciortino, Emilia Turco, Paola Defilippi, Enzo Calautti, Sara Cabodi

**Affiliations:** Department of Biotechnology and Health Science, Molecular Biotechnology Center, Università di Torino, Via Nizza, 52 Torino, Italy

## Correction

Following publication of the original article [1], the authors reported an error in the name of the 11th author. The author’s name was incorrectly published as “Vincenzo Calautti”, instead of “Enzo Calautti”.

The publishers apologise for this error. The original article [1] has been updated.
