# Supplementary material for: Conditional ablation of p130Cas/BCAR1 adaptor protein impairs epidermal homeostasis by altering cell adhesion and differentiation
Source: Cell Commun Signal. 2018 Nov 3;16:73. doi: 10.1186/s12964-018-0289-z (PMC6215608; doi:10.1186/s12964-018-0289-z)
Supplement: Supplementary file 1 — Figure S1. Skin specific deletion of p130Cas/BCAR1 gene. Figure S2. Keratin1 staining. Figure S3. Dot plot quantification of Ki67 and PCNA. Fig. S4 ΔNp63 and YAP expression. Figure S5. Epidermal PCNA, YAP and ΔNp63 expression in WT and p130CasKO mice. Figure S6. Dot plot quantification of loricrin and filaggrin staining. Figure S7. Deletion of p130Cas does not alter expression of β1 and β4 integrins. Figure S8. Epidermal p130Cas loss alters E-cadherin expression and barrier formation. (DOCX 6794 kb) [file 12964_2018_289_MOESM1_ESM.docx]

**Additional file 1
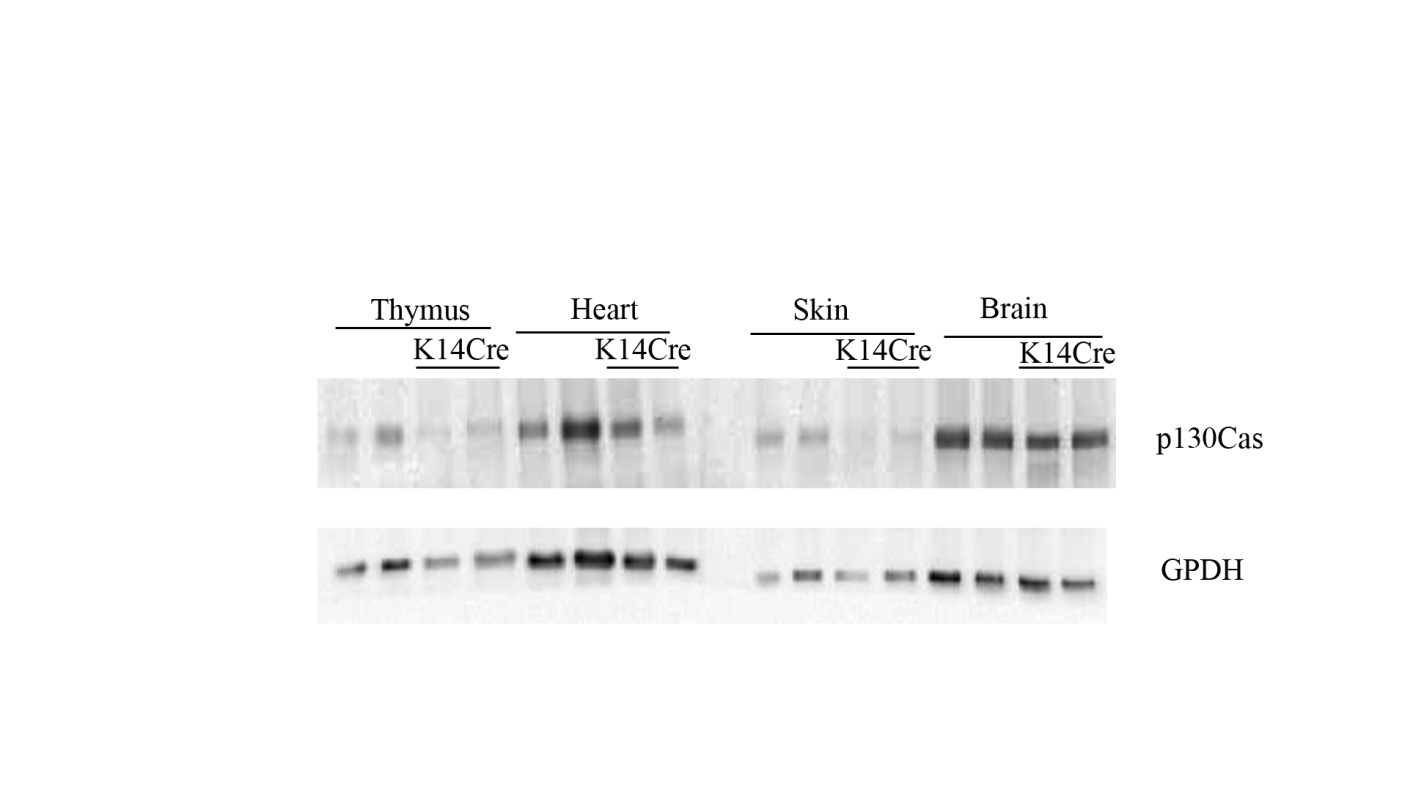
**

**Figure S1. Skin specific deletion of p130Cas/BCAR1 gene**

Different tissues isolated from WT and p130CasKO (K14Cre) were lysed and cell extracts were subjected to western blotting protein analysis to evaluate p130Cas expression.

**
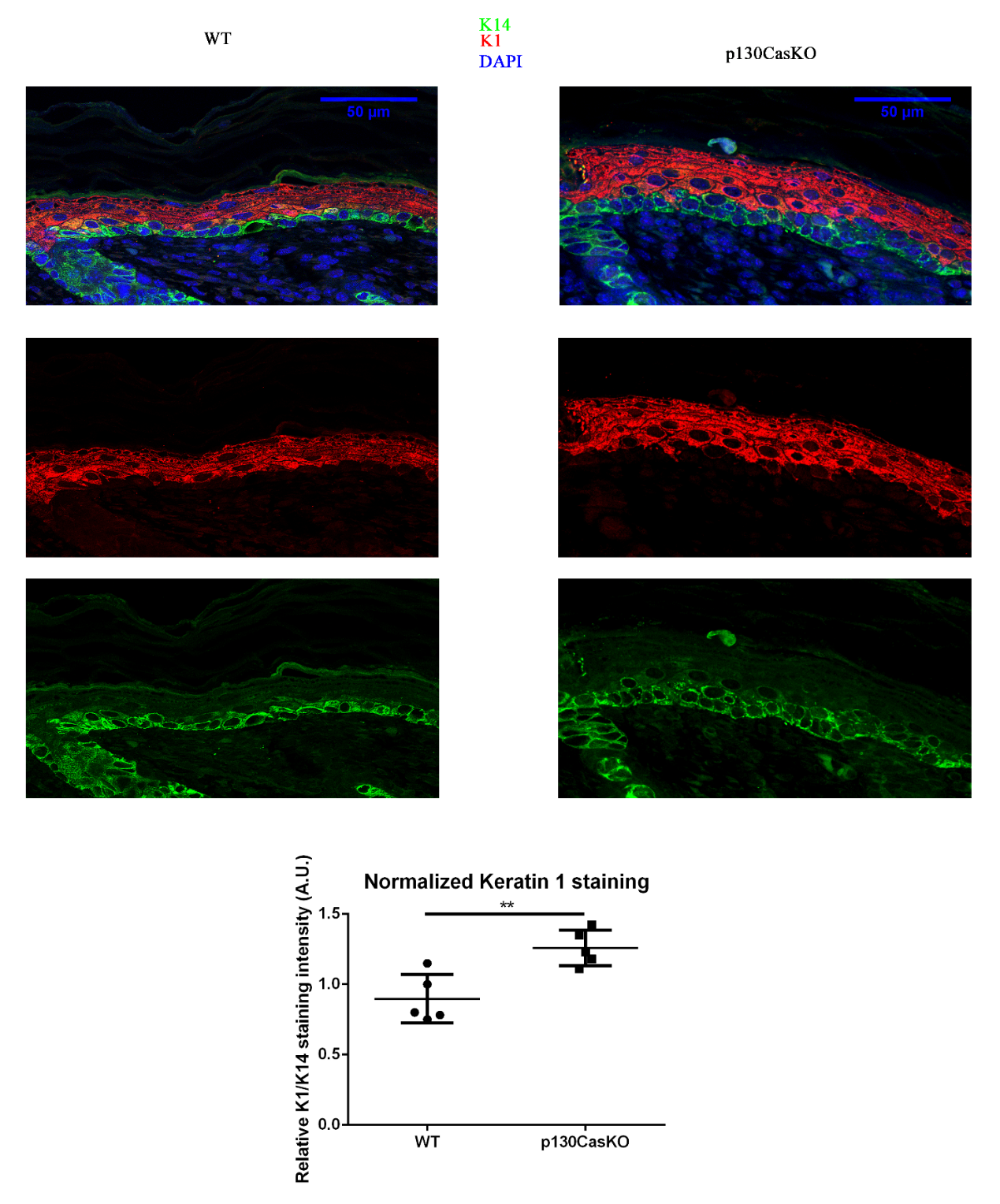
**

**Figure S2. Keratin1 staining**

Representative images of K14 (green) and K1 (red) fluorescence staining of WT and p130CasKO 3-day-old pups skin (40X) with relative single channel images.

**



**

**Figure S3. Dot plot quantification of Ki67 and PCNA**

Dot plots of the quantification of Ki67 (left panel) and PCNA staining (right panel) in WT and p130CasKO as shown in Figure 2A and C, respectively (field equal to 350 μm). The data represented the mean ± S.D. from 10 WT and 10 p130CasKO mice (*p<0.05).

**A**

**

**

**B**

**

**

**Figure S4. ΔNp63 and YAP expression**

**(a)** Paired analysis of ΔNp63 expression (Tubulin as loading control) at different times of calcium-induced differentiation. Densitometric analysis of protein levels of at least three independent experiments is shown (*p<0.05, p**<0.01, ***p<0.001).

**(b)** Densitometric analysis of YAP protein expression levels (GAPDH as loading control) of at least three independent experiments is shown.


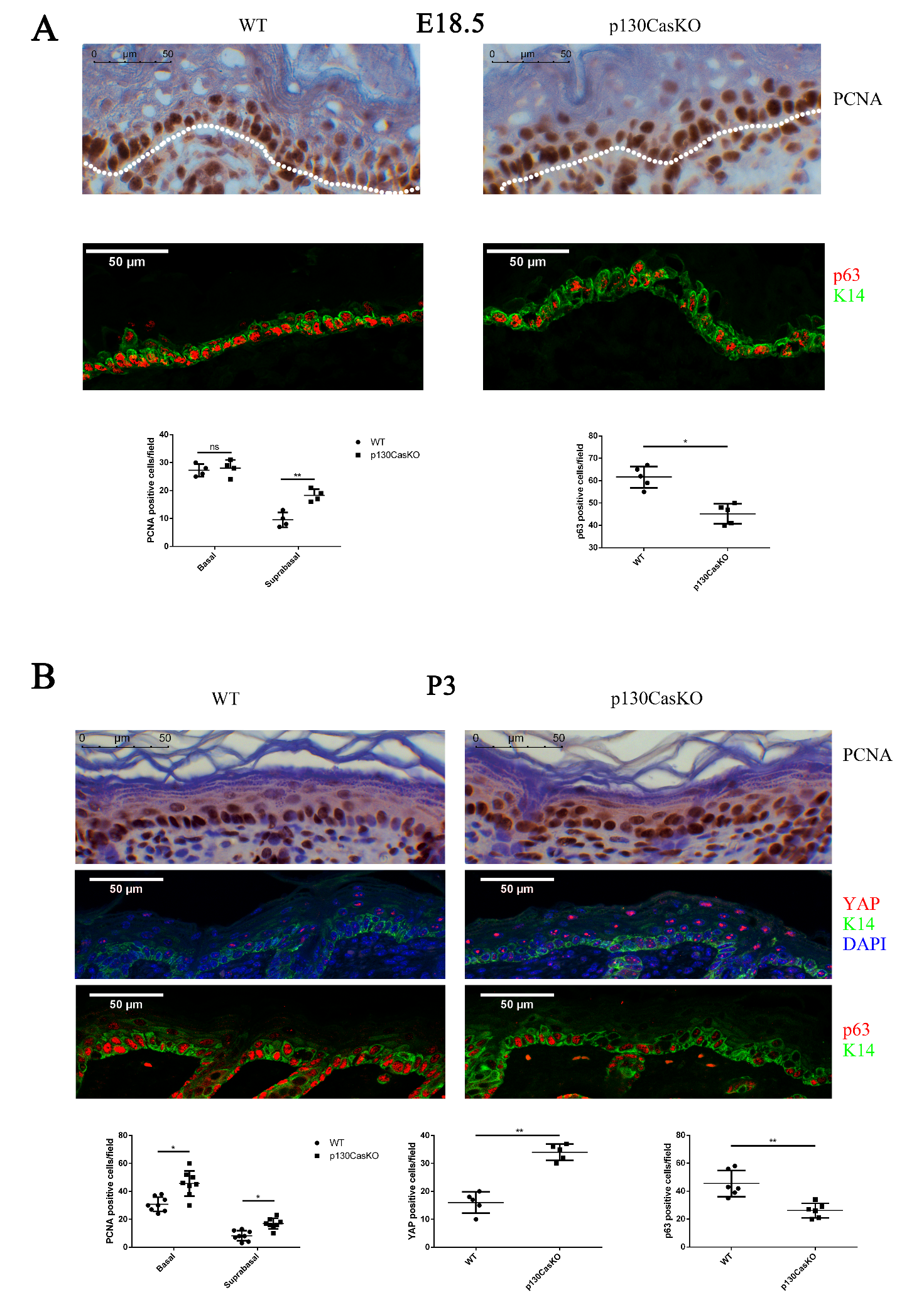


**Figure S5. Epidermal PCNA, YAP and ΔNp63 expression in WT and p130CasKO mice.**

**(a)** Representative images of PCNA immunohistochemical and ΔNp63 immunofluorescent stainings from WT and p130CasKO 18.5 embryos (40X). Quantification of basal and suprabasal PCNA staining is shown in the left panel. Data represented the mean ± S.D. from 8 WT and 8 p130CasKO mice (**p<0.01) (field equal to 200 μm). Quantification of ΔNp63 immunofluorescent staining is shown in right panel. The data represented the mean ± S.D. from 6 WT and 6 p130CasKO mice (*p<0.05) (field equal to 200 μm).

**(b)** Representative images of PCNA immunohistochemical and YAP and ΔNp63 immunofluorescent stainings from WT and p130CasKO 3-days old pups (40X). Quantification of basal and suprabasal PCNA staining is shown in the left panel. The data represented the mean ± S.D. from 8 WT and 8 p130CasKO mice (*p<0.05) (field equal to 200 μm). Quantification of YAP and ΔNp63 immunofluorescent stainings is shown in central and right panels, respectively. The data represented the mean ± S.D. from 6 WT and 6 p130CasKO mice (**p<0.01) (field equal to 200 μm).

**



**

**Figure S6.** **Dot plot quantification of loricrin and filaggrin staining**

Dot plots of the quantification of filaggrin (left panel) and loricrin fluorescence staining of WT and p130CasKO 3-day-old pups skin as shown in Figure 3A and B, respectively. Loricrin and filaggrin staining intensities were normalized on K14 staining intensity, data are represented as mean ± S.D. (*p<0.05, **p<0.01).

**
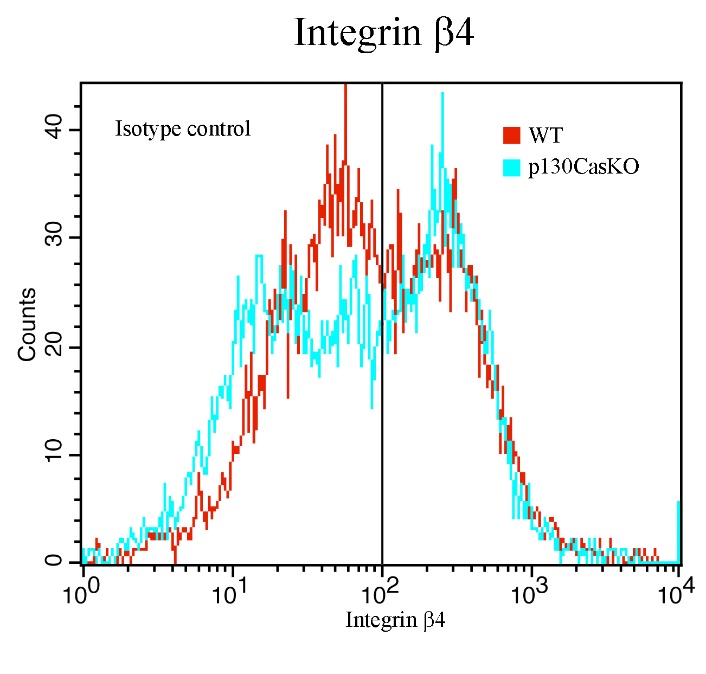

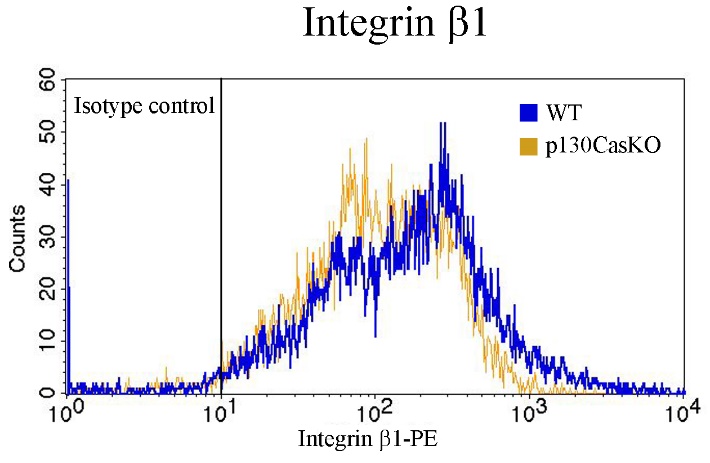
**

**Figure S7.** **Deletion of p130Cas does not alter expression of β1 and β4 integrins**

Mouse primary keratinocytes isolated from WT and p130CasKO mice were analyzed for β1 (left) and β4 (right) integrin expression by flow cytometry.

For β1 staining, cells were incubated with anti β1 antibody (PE anti-mouse/rat CD29 Antibody, BioLegend #102207) or isotype control and directly analyzed by flow cytometry.

For β4 staining, cells were fixed in 4%PFA for 15 minutes and permeabilized with 0.5% Triton X-100 in PBS before incubation with primary anti β4 antibody (homemade rabbit polyclonal antibodies raised against intracellular domain of murine β4 integrin) or isotype control. Staining was revealed by incubation with fluorescent secondary antibodies (Goat anti-Rabbit IgG (H+L) Alexa Fluor 488, ThermoFisher Scientific #R37116) followed by flow cytometry analysis.

Flow cytometric analyses were carried out on a FACSCalibur using CellQuest Software (Becton Dickinson).

**A**


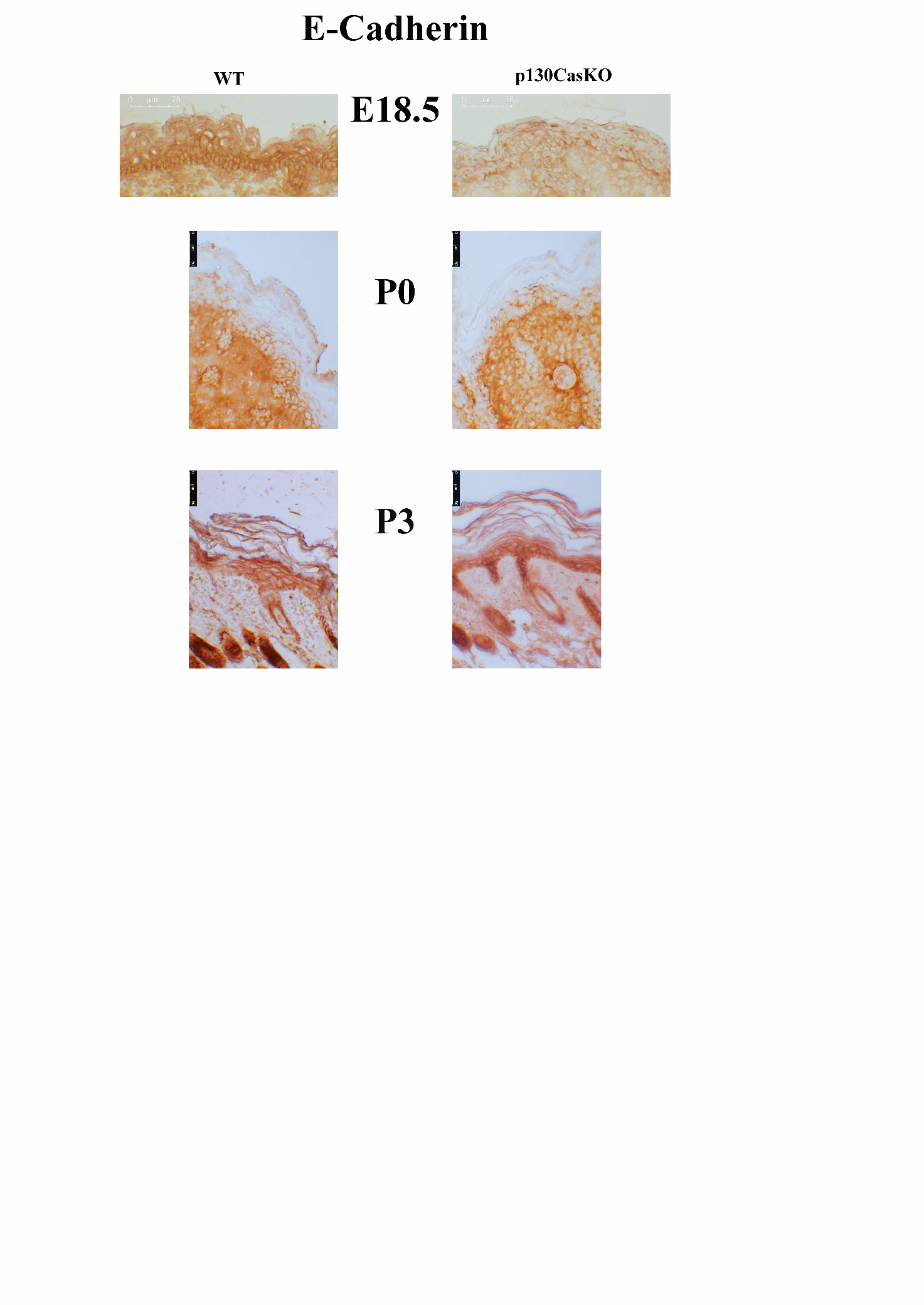


**B**

**
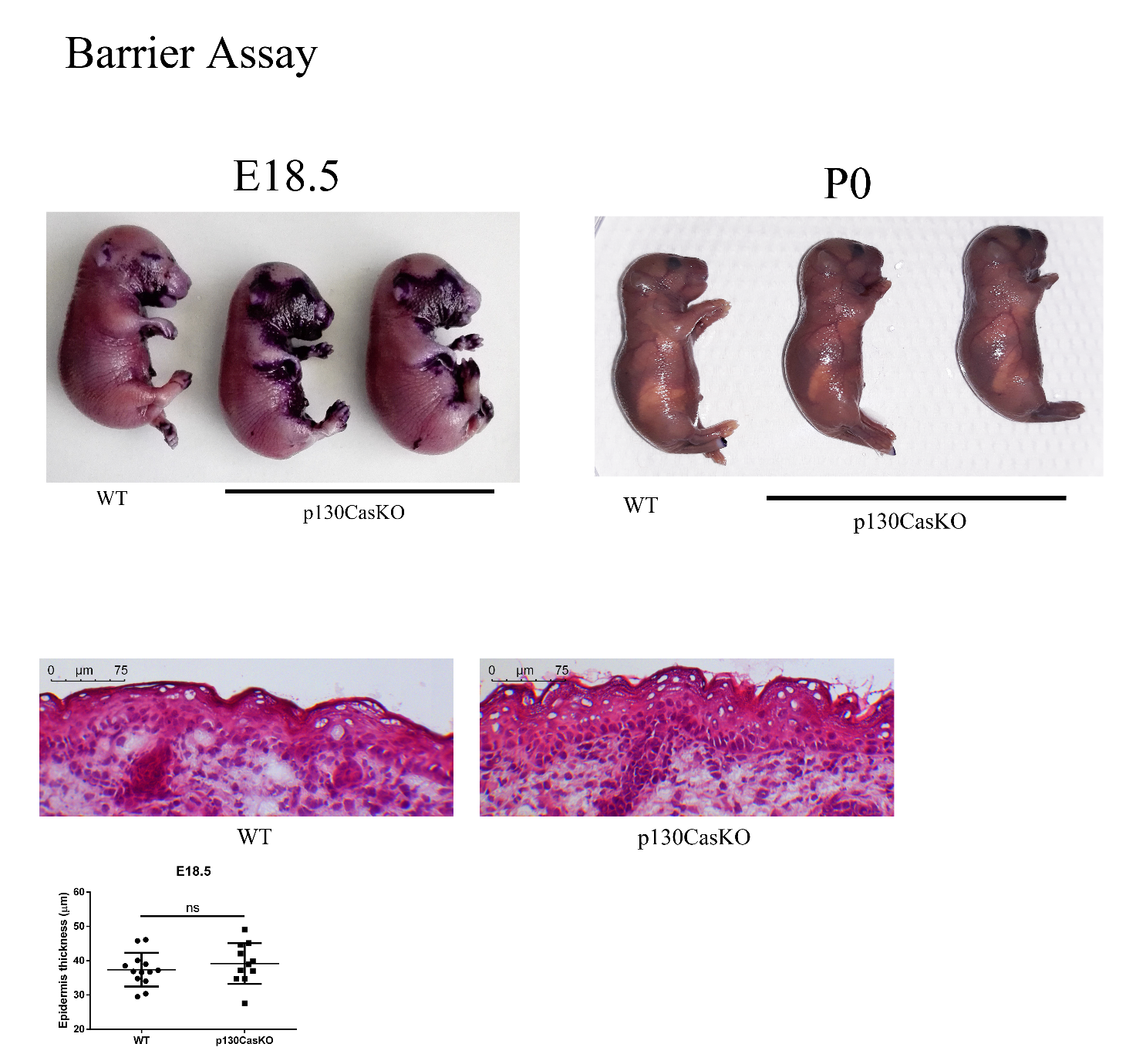
**

**Figure S8.** **Epidermal p130Cas loss alters E-cadherin expression and barrier formation.**

**(a)** Representative images of E-cadherin staining from WT and p130CasKO E18.5 embryos (20X). Scale bar are shown in white.

**(b)** WT and p130CasKO E18.5 embryos were subjected to epidermal barrier assay to evaluate epidermal barrier formation as described in (Schmitz et al. J Invest Dermatol. 2015). Incomplete epidermal barrier formation was detected in approximately 50% of E18.5 p130CasKO embryos.
